# Understanding the Role of Sex Hormones in Cardiovascular Kidney Metabolic Syndrome: Toward Personalized Therapeutic Approaches

**DOI:** 10.3390/jcm13154354

**Published:** 2024-07-25

**Authors:** Mustafa Guldan, Selen Unlu, Sama Mahmoud Abdel-Rahman, Laşin Ozbek, Abduzhappar Gaipov, Andreea Covic, Maria José Soler, Adrian Covic, Mehmet Kanbay

**Affiliations:** 1 Department of Medicine, Koç University School of Medicine, 34450 Istanbul, Turkey; mguldan17@ku.edu.tr (M.G.); sunlu20@ku.edu.tr (S.U.); srahman20@ku.edu.tr (S.M.A.-R.); lozbek18@ku.edu.tr (L.O.); 2 Department of Medicine, School of Medicine, Nazarbayev University, Astana 010000, Kazakhstan; abduzhappar.gaipov@nu.edu.kz; 3 Department of Nephrology, Grigore T. Popa University of Medicine, 700115 Iasi, Romania; accovic@gmail.com; 4 Nephrology Department, Vall d’Hebron University Hospital, Vall d’Hebron Institute of Research, 08035 Barcelona, Spain; mjsoler01@gmail.com; 5 Centro de Referencia en Enfermedad, Glomerular Compleja del Sistema Nacional de Salud de España (CSUR), RICORS2040 (Kidney Disease), 08003 Barcelona, Spain; 6 GEENDIAB (Grupo Español de Estudio de la Nefropatía Diabética), 39008 Santander, Spain; 7 Division of Nephrology, Department of Medicine, Koç University School of Medicine, 34450 Istanbul, Turkey; mkanbay@ku.edu.tr

**Keywords:** sex hormone, chronic kidney disease, metabolic syndrome, cardiovascular kidney metabolic syndrome, diabetes mellitus

## Abstract

Cardiovascular kidney metabolic (CKM) syndrome represents a complex interplay of cardiovascular disease (CVD), chronic kidney disease (CKD), and metabolic comorbidities, posing a significant public health challenge. Gender exerts a critical influence on CKM syndrome, affecting the disease severity and onset through intricate interactions involving sex hormones and key physiological pathways such as the renin–angiotensin system, oxidative stress, inflammation, vascular disease and insulin resistance. It is widely known that beyond the contribution of traditional risk factors, men and women exhibit significant differences in CKM syndrome and its components, with distinct patterns observed in premenopausal women and postmenopausal women compared to men. Despite women generally experiencing a lower incidence of CVD, their outcomes following cardiovascular events are often worse compared to men. The disparities also extend to the treatment approaches for kidney failure, with a higher prevalence of dialysis among men despite women exhibiting higher rates of CKD. The impact of endogenous sex hormones, the correlations between CKM and its components, as well as the long-term effects of treatment modalities using sex hormones, including hormone replacement therapies and gender-affirming therapies, have drawn attention to this topic. Current research on CKM syndrome is hindered by the scarcity of large-scale studies and insufficient integration of gender-specific considerations into treatment strategies. The underlying mechanisms driving the gender disparities in the pathogenesis of CKM syndrome, including the roles of estrogen, progesterone and testosterone derivatives, remain poorly understood, thus limiting their application in personalized therapeutic interventions. This review synthesizes existing knowledge to clarify the intricate relationship between sex hormones, gender disparities, and the progression of CVD within CKM syndrome. By addressing these knowledge gaps, this study aims to guide future research efforts and promote tailored approaches for effectively managing CKD syndrome.

## 1. Introduction

Cardiovascular kidney metabolic (CKM) syndrome stands out as a significant concept that underscores the interrelation between cardiovascular disease (CVD), chronic kidney disease (CKD), and metabolic comorbidities [1]. Highlighting the prevalence and severity of CVD, recent findings from the National Health and Nutrition Examination Survey (NHANES) covering data from 2017 to March 2020 are striking. They indicate that nearly half of adults aged 20 and above, totaling around 127.9 million individuals, are affected by CVD, underscoring the urgent need for attention to this condition [2]. Moreover, CVD retains its status as the leading cause of death, which is particularly poignant as it accounts for 43.6% of all fatalities among individuals with end-stage renal disease (ESRD). These statistics underscore the critical importance of holistic approaches like CKM syndrome in addressing the complex interplay between cardiovascular health, kidney function, and metabolic factors for effective medical intervention [2].

Extensive evidence from observational studies and clinical trials shows the frequent coexistence of metabolic syndrome and cardiovascular and renal diseases. In line with these findings, the American Heart Association introduced CKM syndrome, which would allow a more holistic approach to these diseases that are intricately related in their pathogenesis as well [1]. Various hormonal, neural, and hemodynamic interrelated processes play a role in the pathogenesis of CKM syndrome [3]. The hyperglycemic state in metabolic syndrome induces oxidative stress and increases reactive oxygen species (ROS) production, which provokes organ damage through the activation of polyol and hexosamine pathways. The resulting protein kinase C (PKC) activation and advanced glycation end-product (AGE) formation cause direct damage to the renal and cardiovascular systems by increasing tissue stiffness as well as inducing proinflammatory and profibrotic signaling pathways [2,4]. Furthermore, the local renin–angiotensin system (RAS) is activated in the cardiovascular and renal systems in hyperglycemic states, which provokes vasoconstriction, resulting in increased tissue fibrosis and dysfunction [5]. Apart from the increased ROS production and RAS activation, intensified endoplasmic reticulum (ER) stress and disturbed calcium metabolism are involved in cardiovascular and renal damage through cardiomyocyte apoptosis, atherosclerosis, and diabetic kidney disease (DKD) progression, to name but a few [2].

Gender emerges as a pivotal factor influencing the severity and onset of CKM syndrome [2] due to variations in sex hormone levels and their intricate interactions with key physiological processes, such as the RAS, oxidative stress [6], inflammation [7], vascular disease [8], and insulin resistance [9], that are shown to play a crucial role in the pathogenesis. For instance, while CVD tends to occur less frequently in women compared to men, the outcomes can be more dire for women, with higher mortality rates and poorer prognoses following acute cardiovascular events [10]. Interestingly, a noticeable gender-related disparity exists regarding the treatment of kidney failure via dialysis, with a higher frequency observed in men. This contrasts with the prevalence of CKD in the general population, where women exhibit higher rates [11].

The current landscape of CKD research is marked by a notable scarcity of large-scale investigations, with gender-specific considerations often being overlooked in the customization of CKD treatments, posing challenges to achieving optimal patient outcomes. The intricate pathophysiological mechanisms contributing to the gender disparities in CKD remain elusive, and the nuanced roles of estrogen, progesterone, and testosterone derivatives in the pathogenesis of CKD have yet to be fully elucidated, hampering their integration into clinical evaluations and therapeutic decision-making processes. To address these shortcomings, there is a pressing need for a paradigm shift toward developing personalized treatment strategies tailored to the multifaceted nature of CKD syndrome.

Our objective is to summarize the intricate relationship between sex hormones, gender disparities, and the progression of CVD, along with the potential underlying mechanisms driving this phenomenon. Such a comprehensive review is paramount due to the fragmented and disorganized nature of existing knowledge in this domain. By consolidating and synthesizing this information, we aim to enhance our understanding of this facet of CKD syndrome and provide a foundation for future clinical investigations.

## 2. Sex Hormones and Renal Health

Tailored treatment modalities in CKD are pivotal for enhancing the treatment efficacy and success in nephrological conditions. Gender disparities emerge as one of the most influential factors, which represents an important step toward personalized CKD treatments [12]. Numerous studies have indicated a higher prevalence of CKD in the female population compared to males [13]. A meta-analysis shows that the pooled prevalence of CKD stands at 13.0% (95% CI 11.3–14.9) in females and 12.1% (95% CI 10.3–14.1) in males, resulting in a pooled female-to-male prevalence ratio of 1.07 (95% CI 0.99–1.17) [14]. However, recent research stresses that although CKD is more prevalent among women, a larger proportion of men receive nephrological care compared to women [11]. Although the exact reason for this phenomenon is still unclear, the faster progression of CKD in men could be a potential cause as it would require them to have more outpatient visits. This difference in the progression rates could be due to biological factors or socioeconomic and lifestyle differences. Nevertheless, this is far from being a complete explanation of the discrepancy between the two sexes, and more research is needed [15]. In a comprehensive study involving 3939 adults with CKD, women demonstrated a significantly reduced likelihood of progressing to ESRD (1.5 times lower than men) and mortality, alongside diminished risks of other CKD-related outcomes, such as a 50% decline in the estimated glomerular filtration rate (eGFR) and progression to CKD stage 5 compared to men [16]. In adults with CKD, women exhibit significantly lower risks of cardiovascular events, cardiovascular mortality, and overall mortality compared to men, with the hazard ratios ranging from 0.55 to 0.76 across various outcomes (*p* < 0.001) [17]. Consequently, despite having a lower prevalence compared to females, the prognosis among male patients is poorer [18]. The Korean National Health and Nutrition Examination Survey study reports a prevalence of CKD of 8.9%, with 7.4% in men, 4.7% in premenopausal women, and a notably higher 20.1% in postmenopausal women, providing a strong indicator of estrogen’s protective effect [19]. Various mechanisms could play a role in the renoprotective role of estrogen, as will be explained in more detail in the following sections. Estrogen affects the synthesis, function and degradation of matrix components, cytokines, ROS and vasoactive agents, which have a cumulative effect on kidney function and metabolism [15]. Understanding the background pathophysiology is crucial for planning treatments according to diverse clinical profiles.

### 2.1. Oxidative Stress and Inflammation

Oxidative stress and inflammation play pivotal roles in the complex interplay between gender and kidney health. CKD is a low-grade inflammatory process, as shown by the correlation between the degree of renal impairment and the levels of various markers of inflammation, such as C-reactive protein (CRP), interleukin-6 (IL-6), and tumor necrosis factor alpha (TNF-α) [20,21]. Studies indicate that peripheral polymorphonuclear leukocytes and CD14+/CD16+ cells mediate this chronic inflammatory state in CKD and provoke the further progression of renal damage by participating in atherosclerotic and uremic processes. The increase in oxidative stress is suggested to play a role in the enhanced inflammatory state of CKD patients. In fact, ROS can activate transcription factors regulating inflammatory gene expression, such as nuclear factor kappa B (NF-κB) [22]. In line with these findings, studies in CKD patients show that oxidative stress markers such as plasma 8-isoprostanes (8-epiPGF2a), 15-F(2t)-isoprostane, malonyldialdehyde, and serum total antioxidant status (TAS) increase as the glomerular filtration rate deteriorates [23,24,25]. A study in CKD patients also found a correlation between the oxidized LDL and CRP levels, further strengthening the link between inflammation and oxidative stress in the disease process [25]. These findings suggest that oxidative stress and inflammation have a direct role in the progression of renal injury. However, clinical studies do not focus on the comparison of renal oxidative damage in different genders. Therefore, experimental animal studies are needed to understand the effect of sex hormones on this process. In a mouse model of acute renal ischemia–reperfusion injury, males exhibit increased susceptibility, characterized by elevated levels of inflammatory cytokines such as tumor necrosis factor-alpha, monocyte chemotactic protein-1, interferon-gamma, and chemokine (C-C motif) ligand 17, with testosterone exacerbating these effects and estrogen ameliorating them [26]. Females tend to show preserved kidney function and less severe ischemic acute renal failure attributed to elevated nitric oxide (NO) levels, respectively, leading to reduced peroxynitrite levels compared to men. This suggests a potential protective effect against oxidative and nitrosative stress in females [27].

**Figure 1:**
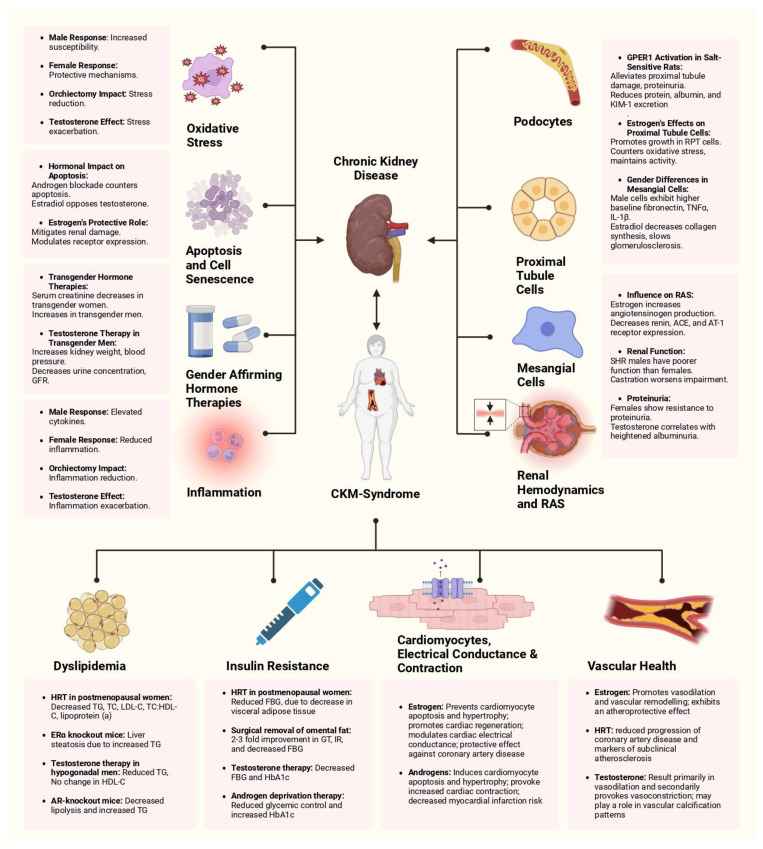
Summary of the existing evidence on the interplay between CKM syndrome, renal health, and sex hormones. This figure illustrates the multifaceted impact of the sex hormones, estrogen and testosterone, and different hormonal therapies on components of CKM syndrome, encompassing renal function, cardiovascular health, and metabolic regulation, with a greater focus on chronic kidney disease and individual mechanisms within the kidney. It contrasts male and female responses to hormonal changes, generally emphasizing the protective mechanisms in females and increased susceptibility in males. Abbreviations: GFR: glomerular filtration rate; GPER1: G protein-coupled estrogen receptor 1; KIM-1: kidney injury molecule 1; RPT: renal proximal tubule; TNFα: tumor necrosis factor-alpha; IL-1β: interleukin-1 beta; ACE: angiotensin-converting enzyme; AT-1 receptor: angiotensin II receptor type 1; SHR: spontaneously hypertensive rats; TG: triglycerides, TC: total cholesterol, LDL-C: low-density lipoprotein cholesterol, HDL-C: high-density lipoprotein cholesterol; FBG: free blood glucose; GT: glucose tolerance; IR: insulin resistance.

Conversely, orchiectomy in male mice significantly reduces post-ischemic oxidative stress and kidney injury, accompanied by an increase in the expression and activity of manganese superoxide dismutase (MnSOD), highlighting its critical role [28]. Furthermore, male mice exhibit noticeable kidney dysfunction and extensive proximal tubular damage in the outer cortex (S1 and S2 segments) upon tunicamycin-induced endoplasmic reticulum (ER) stress [29]. In contrast, female mice demonstrate milder kidney injury primarily localized to the proximal tubules in the inner cortex (S3 segment), without experiencing a decline in kidney function [29]. Interestingly, administering testosterone to female mice before tunicamycin exposure leads to a phenotype resembling that of male mice, including a concurrent decline in renal function, tissue morphology alterations, and induction of ER stress markers [29] (Figure 1).

### 2.2. Apoptosis, Cell Senescence, and Renal Aging

The role of apoptosis, cell senescence, and aging in the development of kidney disease demonstrates gender variability. The anatomic and physiological changes during the aging process are defined as “senescence”, which is separate, albeit difficult to differentiate, from the changes induced by pathological conditions. An increased number and size of renal cysts, focal scars, decreased cortical volume, increased cortical surface roughness, increased medullary volume and increased atherosclerosis of the renal arteries are among the macroscopic changes in the renal aging process. The microscale changes, on the other hand, are increased glomerulosclerosis, tubular atrophy, interstitial fibrosis and arteriosclerosis [30]. Mitochondrial oxidative stress and damage are suggested to play a key role in this process by affecting podocyte injury, apoptosis and glomerulosclerosis [31,32].

Many experimental animal studies suggest a key role of sex hormones in the control of apoptosis in kidneys. Blocking the androgen receptor prevented testosterone-induced apoptosis, while estradiol (E2) treatment countered this effect. Testosterone treatment increased the levels of apoptotic proteins such as Fas, FasL, and Bax, while decreasing the levels of the anti-apoptotic protein Bcl-2, alongside activation of caspase-3 and cleavage of PARP-1, indicating the increased vulnerability of renal tubular cells to apoptosis in men with chronic renal diseases [33,34]. Gender differences are evident in the severity of renal injury and tissue remodeling, with male rats exhibiting higher expression of markers like vimentin and proliferating cell nuclear antigen (PCNA), which correlate with more significant renal damage [35]. Mitochondrial enzyme activity, particularly vimentin and translocator protein (TSPO), is pronounced in the proximal tubules of male rats after reperfusion, suggesting the greater severity of renal injury in males regardless of hormonal influences [35].

Additionally, estrogen plays a protective role in mitigating age-related renal injury and oxidative stress, potentially through the modulation of angiotensin II receptor expression and other molecular pathways [36]. Estrogen receptor (ER) alpha (ERα) expression in podocytes mediates protection against apoptosis, both in vitro and in vivo [37,38]. Testosterone and 17β-estradiol exhibit contrasting impacts on podocyte apoptosis preceding glomerulosclerosis in female mice lacking estrogen receptors [39]. Finally, testosterone-induced apoptosis in kidney tubule cells suggests a potential mechanism of gender differences in the progression of renal disease [40] (Figure 1).

### 2.3. Transgender Therapies and Implications for Kidney Health

Transgender hormone therapies provide a real-world example supporting the hypothesis regarding the influence of sex hormones on kidney health in individuals with gender variance. Gender-affirming hormone therapies (GAHTs) are composed of transdermal or injectable testosterone administration for transgender men, and transdermal, injectable, oral or sublingual estradiol and antiandrogen therapy for transgender women [41]. In transgender individuals undergoing GAHT, significant changes in the serum creatinine levels occur over time. Specifically, in transgender women, the serum creatinine levels decrease by −0.07 at 6 months and by −0.09 at 12 months, while in transgender men, they increase by 0.14 at 3 months, 0.21 at 6 months, and 0.15 at 12 months [42]. These findings are supported by a meta-analysis involving 488 transgender men and 593 transgender women, indicating a noteworthy shift in the serum creatinine levels after 12 months of GAHT [41]. The changes in the serum creatinine in individuals receiving GAHT are highly influenced by the changes in lean muscle mass, altering the estimated glomerular filtration rate (eGFR) equations. However, alterations in body composition cannot be solely held accountable for the changes in the serum creatinine, which are probably also influenced by the changing GFR as well. Nevertheless, the effect of GAHT on the GFR is currently not well elucidated in clinical studies and the influence of GAHT on other kidney function markers, such as cystatin C, albuminuria and proteinuria, remains uncertain [41]. Current calculations of the eGFR remain inadequate for the measurement of kidney functions in transgender individuals, and more studies are needed to determine better outcome measures in this population.

It is challenging to determine the mechanisms underlying the effects of exogenous sex hormone administration on kidney function in humans due to the longevity of studies and the difficulty of differentiating sex and gender in prognostic studies [43]. Nevertheless, experimental animal studies suggest some possible mechanisms concerning the effect of GAHT on kidney function. Research on testosterone therapy, used for gender affirmation in transgender men, has shown changes in the renal structure and function in female rats. These changes include increased kidney weight, reduced urine concentration, decreased GFR, enlarged glomerular size, and elevated blood pressure compared to controls [44]. Furthermore, a study in male rats on the hormone therapy used by transgender women showed reduced body weight with intact blood pressure, increased plasma concentrations of urea and creatinine as well as increased excretion of sodium, potassium and increased urinary volume [45]. The alterations provoked by hormone therapy in the RAS, sympathetic nervous system activation, and epithelial sodium channel (ENaC) expression may play a role in changing the blood pressure and plasma sodium concentration. Furthermore, exogenous hormone administration alters the endothelin and nitric oxide (NO) quantities, influencing the level of vasoconstriction and thus the blood pressure [46,47]. These mechanisms, and potentially other additional factors, play a role in the occurrence of renal injury in transgender individuals. All in all, these findings underscore the complex interplay between sex hormones and kidney health in transgender individuals undergoing hormone therapy, suggesting potential implications for clinical management and the need for further investigation of the effects of hormonal treatments on renal function (Figure 1).

### 2.4. Renal Hemodynamics and Gender Disparities

Gender disparities play a pivotal role in hemodynamic processes, affecting vasoconstriction, vasodilation, glomerular dynamics, and vasoprotection. Estrogen tends to increase the production of angiotensinogen, the precursor of angiotensin II, especially through oral administration [48], and to decrease the expression of renin, angiotensin-converting enzyme (ACE), and AT-1 receptors, crucial components involved in angiotensin II production and its actions [48]. Additionally, in spontaneously hypertensive rats (SHR), males typically exhibit poorer renal function compared to females, and castration worsens this impairment, underscoring the significant impact of sex hormones on renal well-being [49]. This influence may be mediated by changes in the oxidative stress and activity within the renin–angiotensin system, as evidenced by alterations in the ACE and ACE2 levels.

Another study illustrates the significant role of Endothelin-1 (ET-1) receptors in facilitating vasodilation among young women, although this effect wanes post-menopause, leading to compromised vasodilatory function in postmenopausal women [50]. Additionally, male rats display heightened susceptibility to proteinuria induced by mild chronic inhibition of nitric oxide synthase (NOS) in comparison to females [51]. Females demonstrate resistance to the development of proteinuria at doses of 20 and 30 mg/L L-NNA, while males exhibit notable increases in the proteinuria levels (139 +/− 35 mg/day and 318 +/− 82 mg/day, respectively, compared to 55 +/− 11 mg/day in controls), implying a protective role of estrogen in females and an exacerbating effect of androgens in males [51]. Elevated testosterone levels are also correlated with heightened albuminuria, coupled with a reduction in the number of glomeruli in mouse models, suggesting the potential involvement of testosterone in modulating renal function [52] (Figure 1).

### 2.5. Functional and Structural Effects of Sex Hormones on Nephrons

Sex hormones like estrogen have significant effects on the structural and functional aspects of nephrons beyond just molecular mechanisms. For instance, in female rats with reduced kidney function, estrogen treatment notably decreased the indicators of renal damage such as albuminuria and glomerulosclerosis [53]. This treatment also reduced the expression of markers associated with podocyte injury and systems causing damage to the glomeruli, indicating estrogen’s substantial role in protecting the kidneys. In another study involving a model of type 2 diabetic kidney disease, administering 17 beta-estradiol and tamoxifen resulted in reduced levels of albumin in the urine, decreased glomerular volume, and less accumulation of extracellular matrix [54]. These effects are likely due to the protection of podocytes from injury, as shown by the lower levels of podocyte transforming growth factor-beta mRNA and higher expression of estrogen receptor subtype beta protein [54].

The Dahl salt-sensitive (SS) rat is a genetic rodent model of salt-sensitive hypertension that rapidly develops decreased renal function with increased proteinuria when challenged with a high-salt (HS) diet. There is also a Dahl salt-resistant strain that does not develop a considerable increase in blood pressure upon consuming a HS diet and is used as a normotensive control [55]. Activation of GPER1 in female Dahl SS rats on a HS diet has been shown to alleviate damage to the proximal tubules and reduce proteinuria. This was evidenced by a decrease in the urinary excretion of protein, albumin, and kidney injury molecule 1 (KIM-1), which serves as an indicator of proximal tubule impairment [56]. Additionally, the effects of estrogen, including 17 beta-estradiol and alpha estradiol, on primary rabbit kidney proximal tubule (RPT) cells were investigated under hormone-free conditions, revealing growth-promoting effects even at low concentrations [57]. Specifically, 17 beta-estradiol, but not progesterone or testosterone, countered the decrease in the alpha-methyl-D-glucopyranoside (alpha-MG) uptake induced by tert-butyl hydroperoxide (t-BHP) in renal proximal tubular cells, indicating its protective role against oxidative stress in proximal tubule activity [58,59].

Male rat mesangial cells exhibit naturally higher baseline levels of fibronectin, TNF- α, and interleukin-1 beta (IL-1β) compared to female cells, which may play a role in the quicker advancement of chronic renal disease in males [60]. Furthermore, sex hormones are known to influence these differences. Interestingly, E2 has been found to significantly decrease the overall collagen synthesis, including collagen types I and IV, potentially contributing to the slower progression of glomerulosclerosis in females. In contrast, testosterone has minimal effects on proliferation and does not impact mesangial collagen synthesis [61,62] (Figure 1).

### 2.6. Sex Hormones and Cardiac Health

The effect of estrogen on cardiac function and metabolism has been extensively studied in humans and various animal models, showing an intricate relationship. Estrogen exerts its effect on the heart through three main receptors: ERα, ERβ, and G-protein-coupled estrogen receptor (GPER) [63]. GPER is a transmembrane receptor, whereas ERs are found in the cytosol, nucleus, and mitochondria of cardiomyocytes, although ERα was demonstrated to be present on the cardiomyocyte plasma membrane as well [64,65]. ERs can function as ligand-activated transcription factors, termed the “genomic effect of ERs”. They can also regulate cell regulatory proteins such as GPER or work as plasma membrane proteins themselves, which is termed as “nongenomic effect of ERs” [66,67].

The effects of androgens on cardiomyocytes and cardiac function have not been studied as extensively as estrogens. Androgens mediate their effects by binding to the androgen receptor in cardiomyocytes. Androgen receptors function as ligand-induced transcription factors, named the “genomic effect”, similarly to ER. Furthermore, membrane-associated androgen receptors regulate the signal transduction cascades by activating L-type calcium channels, named the “nongenomic effect” [68].

### 2.7. Sex Hormones and Cardiac Hypertrophy

Cardiac hypertrophy is an adaptation mechanism of the heart in response to hemodynamic stress and is mainly regulated by cardiac myocytes and fibroblasts, which express estrogen and androgen receptors [69].

Estrogen can inhibit cardiomyocyte hypertrophy through various mechanisms. Studies in rats and mice suggest that estrogen inhibits calcineurin-related hypertrophy by inducing modulatory calcineurin-interacting protein 1 (MCIP1) [70], represses matrix metalloproteinase-2 transcription by activating MAPK [71], modulates mTOR activity [72], activates atrial natriuretic peptide [73] and suppresses pro-hypertrophic histone deacetylase production and activity, whereas it derepresses antihypertrophic histone deacetylase [74]. Furthermore, studies in mice indicate that estrogen regulates physiologic cardiac growth and exercise-induced cardiac hypertrophy [75].

In contrast, androgens have prohypertrophic effects on cardiomyocytes by activating the mTORC1/S6K1 axis, NFAT, and MEF2 through Ca^2+^/calmodulin-dependent protein kinase II (CaMKII) and inhibiting GSK-3β [76,77,78].

The contrasting effects of estrogens and androgens may help explain males’ greater likelihood of developing cardiac hypertrophy after myocardial infarction, as observed in different studies [79,80].

### 2.8. Sex Hormones and Cardiac Bioenergetics

Studies in rats and mice show that estrogen enhances mitochondrial activity and causes increased ATP production in cardiomyocytes while also reducing oxidative stress, possibly through regulating PGC-1α [81,82]. In contrast to estrogen, androgen has pro-oxidative effects on cardiomyocytes. It promotes ROS generation by increasing enzymes such as NAD(P)H oxidases, Xanthine oxidase, and cyclooxygenase 2 (COX-2) [83]. Estrogen and androgens are also suggested to regulate certain glucose transporters to modulate glucose uptake into cardiomyocytes [63].

### 2.9. Sex Hormones and Cardiomyocyte Apoptosis

Estrogen prevents cardiomyocyte apoptosis, but the definite mechanism is not yet known. Nevertheless, mice and rat models have described many intracellular signaling pathways. PI3K/Akt pathway activation, SIRT1 and corticotropin-releasing hormone receptor type 2 upregulation, NF-κB, and ASK1 inhibition can be listed among them [84,85,86,87,88,89]. In contrast, a study in rats showed that androgens induce cardiomyocyte apoptosis in a dose-dependent manner by decreasing the antiapoptotic Akt pathway [90].

### 2.10. Sex Hormones and Cardiac Regeneration

In addition to preventing apoptosis, estrogen can promote cardiac regeneration. In a study conducted on cardiac stem cells isolated from mice, those that were treated with estrogen showed increased production of protective factors, and they improved cardiac function when infused into mice hearts [91]. In another study, estrogen-deficient mice exhibited lower telomerase activity and more DNA damage in the cardiac stem cells, which improved upon estrogen replacement [92].

### 2.11. Sex Hormones and Cardiac Electrical Conductance and Contraction

Estrogen modulates cardiac electrical conductance, thus impacting the cardiac rhythm and contractility. In a dog model with ischemia–reperfusion-induced ventricular arrhythmia, the incidence of lethal ventricular tachyarrhythmias was lower in dogs that received estrogen, suggesting an anti-arrhythmogenic role of estrogen [93]. Furthermore, in estrogen-deficient mice, myocardial contractile function was significantly impaired, showing the importance of estrogen in ensuring harmonious cardiac contractility [94].

Estrogen regulates electrical conductance by modulating myriad ion channels and ion exchangers. A study in rats showed that estrogen stimulates NO release, which inhibits Na^+^/H^+^ exchanger (NHE1) in ischemia–reperfusion injury [95], and another study in dogs showed that estrogen-stimulated NO release increased the opening of Ca^2+^-activated K^+^ (K(Ca)) channels [96]. Furthermore, a study in mice showed that estrogen inhibits L-type Ca^2+^ channels [97]. In contrast, androgens cause an increase in L-type Ca^2+^ channels on the cardiomyocyte surface. Furthermore, by increasing the β1-adrenergic receptor and Na^+^/Ca^2+^ exchanger expression, they provoke increased cardiac contraction [98].

These effects of sex hormones on the cardiac rhythm and contractility may also help explain the differences observed in the surface electrocardiograms of males and females. Sympathetic modulation and autonomic blockage have a more pronounced effect in females, who are more prone to have a faster resting heart rate, lower QRS voltage, shorter QRS, and longer QTc duration, with fluctuations during the menstrual cycle [99]. Furthermore, some studies indicate an arrhythmogenic role of estrogen. In a study, females using oral contraceptive pills were found to be at a higher risk of ventricular ectopy [100]. In other studies, syncope, congenital long QT syndrome, and drug-induced QT prolongation risk were found to be higher in females [101]. Nevertheless, these studies do not indicate a causal relationship, and the effects of estrogen and androgen on cardiac arrhythmias need to be investigated further (Figure 1).

### 2.12. Sex Hormones and Heart Failure

Many studies have shown that males are more likely to have heart failure compared to females, and females have a greater survival rate than males with this condition, despite being older [102]. However, increased androgen levels are not found to be correlated with heart failure. In contrast, observations in prostate cancer patients receiving androgen deprivation therapy (ADT) revealed an increased risk of heart failure in these patients [103,104]. Furthermore, studies in male patients with chronic heart failure showed a decrease in serum dehydroepiandrostenedione sulfate (DHEAS) and E2 as well as an increase in the sex hormone-binding globulin (SHBG) levels [84,105,106]. Some studies found that the derangement levels of these hormones and proteins were in proportion to the severity of chronic heart failure [107,108].

All these results suggest that hormonal imbalances play a role in heart failure occurrence, and heart failure itself may further worsen hormonal imbalances, creating a vicious cycle.

### 2.13. Sex Hormones and Coronary Heart Disease

Studies have shown that females experience their first ischemic heart disease approximately 6–10 years after males, which suggests a protective effect of estrogen against coronary heart disease [109]. Even though clinical trials of postmenopausal hormone replacement therapy (HRT) did not find a beneficial cardiovascular outcome, animal models demonstrate the cardioprotective role of estrogen in ischemia–reperfusion injury [110,111]. A study in rabbits showed that estrogen administration before coronary artery occlusion reduces the infarct size regardless of gender [112]. Studies in mice, rats, and rabbits suggest that estrogen exerts its cardioprotective functions via all three types of ER before and after ischemia, although there are conflicting results between studies [113]. Similarly to the case of heart failure, a decrease in the androgen levels does not lead to decreased myocardial infarction rates in males. In contrast, studies show that ADT increases the myocardial infarction risk [114].

## 3. Sex Hormones and Vascular Health

ERα, ERβ, and GPER are expressed on endothelial cells and vascular smooth muscle cells, controlling their function through genomic and nongenomic pathways [67]. Studies show that their expression can vary with age, sex, and circulating estrogen levels, as ERα expression decreases with menopause and in ovariectomized rats, and GPER decreases with aging [115,116]. Estrogen modulates transcriptomic changes in vascular cells by binding to cytosolic ER. Apart from its genomic roles, estrogen binds to membrane receptors to increase eNOS, which results in the activation of numerous pathways, such as proto-oncogene tyrosine-protein kinase (c-Src), epidermal growth factor receptor (EGFR), phosphoinositide-3-kinase (PI3K), and extracellular-signal-regulated kinase (ERK). This way, estrogen promotes vasodilation by increasing vasodilators such as NO and prostacyclin and by decreasing vasoconstrictors such as angiotensin II and endothelin in the environment [117]. Estrogen decreases the migration and proliferation of vascular smooth muscle cells [118]. Estrogen also promotes vascular remodeling by enhancing matrix metalloproteinase activity [119]. Studies have shown conflicting results regarding estrogen’s effect on arterial stiffness. Some studies in rodents state that estrogen decreases arterial stiffness, whereas others suggest that a lack of estrogen protects against arterial stiffness [120,121].

Similarly to ER, androgen receptors are expressed on endothelial cells and vascular smooth muscle cells of the vasculature [122]. Androgens cross the plasma membrane and bind to cytosolic androgen receptors to exert their genomic actions [123]. Furthermore, the binding of androgens to membrane androgen receptors brings about nongenomic changes in the cells. Membrane androgen receptors have still not been clearly identified, but G protein-coupled receptor family C group 6-member A (GPR6A), Oxoeicosanoid receptor 1 (OXER1), transient receptor potential melastatin 8 (TRPM8) and zinc-regulated transporter [Zrt]- protein (ZIP9) are the potential candidates [124]. The activation of these receptors results in the rapid increase of the intracellular calcium levels as well as the enhancement of the RAS/MEK/ERK MAPK pathway [125]. The interaction of androgens with cytosolic and membrane androgen receptors primarily results in vasodilation. However, apart from their direct effects, androgens interact with multiple physiologic processes to provoke vasoconstriction as well [126] (Figure 1).

### 3.1. Sex Hormones and Hypertension

Studies have shown that in general, males have higher blood pressure compared to females, but this difference disappears in the postmenopausal period, so the prevalence of hypertension in females over 60 years old is greater than in males [127,128]. Studies in rats show that the blood pressure of males is higher compared to their female counterparts. Furthermore, orchiectomy or treatment with testosterone receptor antagonists early in life prevented the later development of hypertension in male rats [129,130].

Clinical trials of postmenopausal HRT failed to show any positive impact [111,131]. Furthermore, even though older males with lower androgen levels tend to have higher blood pressure, androgen replacement worsens hypertension [132].

As explained above, these sex hormones act directly on vascular cells to regulate blood pressure. In addition, they interact with various physiological processes to indirectly control blood pressure. As shown in rats and mice, estrogen increases the RAS components that oppose increased blood pressure, namely ACE2-Ang(1-7)-MasR/AT2R pathway, which was also clinically confirmed with the suppression of vasoconstrictor components upon estrogen therapy in postmenopausal females [133,134]. In contrast, testosterone increases the vasoconstrictor components of RAS, which consist of the ACE-AngII-AT1R pathway [135].

Estrogen modulates the sympathetic nervous system by controlling adrenergic receptor (AR) expression. Studies in rats show that estrogen upregulates β-adrenergic receptors (βAR) and downregulates α-adrenergic receptors (αAR) [136,137]. In contrast, androgens reduce the activity of βAR by interfering with their downstream signaling cascades rather than decreasing their expression in cells [138]. Furthermore, androgens increase the αAR expression in cells. All in all, estrogen enhances β-adrenergic-induced vasorelaxation, whereas androgens promote α-adrenergic-induced vasoconstriction [139].

### 3.2. Sex Hormones and Atherosclerosis

Beyond the lower prevalence of atherosclerotic CVD in premenopausal women compared to age-matched men and the tendency of women to develop more cardiovascular complications post-menopause, all attributed to estrogen-mediated atheroprotection, previous evidence also highlighted sex-specific differences in the plaque characteristics, distribution, and progression [140]. Atherosclerosis is characterized by the complex interplay of the oxidative modification of LDL-C, recruitment of monocytes facilitated by endothelial dysfunction, and subsequent differentiation into macrophages that drive plaque formation through foam cell production and inflammatory cytokine release. This chronic inflammatory process, exacerbated by the postmenopausal estrogen decline in women, underscores the critical role of macrophage polarization dynamics, particularly the impairment of M2 phenotype activation, in influencing the cardiovascular risk progression beyond lipid metabolism alone [141]. ERα mediates atheroprotective effects through both nuclear/transcriptional and membrane-associated pathways, exerting its impact on endothelial cells, macrophages, and miRNA regulation [141,142]. In this context, there has been increasing interest in measuring endogenous sex hormones and exploring their association with atherosclerosis [143,144]. A study following 249 pre- or early peri-menopausal women over a period of up to 9 years found that lower levels of E2 and SHBG, and higher levels of follicle-stimulating hormone (FSH), were independently associated with accelerated progression of subclinical atherosclerosis in women aged 42–57 years [145]. These associations were linked to favorable changes in the lipid profiles, suggesting a potential protective role of estrogen and SHBG against CVD in this population. Despite the recognized risks of HRT in postmenopausal women, the paradoxical protective impact of sex hormones in premenopausal women has garnered attention [140,146]. Therefore, research has not only focused on endogenous sex hormones but also on assessing the effects of HRT on atherosclerosis, with previous studies indicating both positive and neutral outcomes [146,147]. Building upon this research, estrogen replacement therapy has been proposed for investigation in the secondary prevention of atherosclerosis. Most of these studies, however, have not demonstrated any significant benefit in this regard. In this context, the Papworth HRT Atherosclerosis Study found that among postmenopausal women with angiographically proven ischemic heart disease, transdermal HRT did not significantly reduce the rate of primary endpoint events, which were admission to hospital due to myocardial infarction, unstable angina, or cardiac death, with a comparable event rate in the HRT group compared to the control group [148]. Later on, another study involving 1458 postmenopausal women who received 2–3 years of HRT found the severity of atherosclerosis, as indicated by aortic calcification scores, was notably lower in the hormone-treated group compared to non-treated women, with a 30% decrease in all-cause mortality [149]. Additionally, there were significant reductions in cardiovascular and coronary heart disease mortality by 46% and 53%, respectively [149]. A randomized controlled trial involving 180 postmenopausal women (91 receiving 17 beta-estradiol and 89 receiving placebo) found that increased serum levels of estrone, total E2, free E2, and SHBG were significantly associated with reduced progression of carotid artery intima-media thickness, a marker of subclinical atherosclerosis, over a 2-year period [150]. In a double-blind, placebo-controlled trial involving 226 postmenopausal women with coronary artery disease and a mean age of 63.5 years, neither micronized 17β-estradiol alone nor 17β-estradiol plus sequentially administered medroxyprogesterone acetate significantly slowed the progression of coronary-artery atherosclerosis over a median follow-up of 3.3 years. The study found no significant differences in the percent stenosis change between the control group and the estrogen or estrogen–progestin groups, indicating that hormone therapy did not influence atherosclerosis progression in this cohort [151]. Furthermore, it was also proposed that the timing of HRT initiation may influence the outcomes, with suggestions that starting HRT later in the postmenopausal period could potentially lead to adverse cardiovascular outcomes while starting them in recently menopausal women seems favorable [152,153]. During the premenopausal years, estrogen deficiency is a critical factor promoting premature atherosclerosis, which can be mitigated by estrogen-containing contraceptives. In the perimenopausal and early postmenopausal stages, estrogen therapy shows substantial benefits by halting atherosclerosis progression and reducing the mortality risk. However, in the late postmenopausal period, starting estrogen therapy late may potentially increase the coronary heart disease risk due to enhanced plaque inflammatory processes, although this risk could be moderated by prior statin use [154]. GAHTs have also garnered attention in terms of investigating their potential long-term effects and cardiovascular risks in this population. A recent systematic review included 12 studies involving transgender individuals undergoing GAHT and found that GAHT in transgender men was associated with increased markers of subclinical atherosclerosis, such as carotid thickness and pulse wave velocity, while in transgender women, GAHT showed variable effects, potentially decreasing the inflammatory markers and pulse wave velocity but with mixed impacts on arterial stiffness and vasodilation [155]. A study previously demonstrated the potential role of FGF23 in mediating the relationship between sex hormones and CKD. Higher levels of free testosterone are linked to increased FGF-23 in women, while elevated estradiol levels and a higher total testosterone/estradiol ratio correlate with lower FGF-23 in men [143]. Therefore, further research is needed to explore the role of FGF23 as a mediator in this context.

Unlike the extensive body of literature supporting estrogen’s beneficial effects on cardiovascular outcomes and atherosclerosis, testosterone lacks sufficient robust evidence to substantiate its impact in these areas [156]. A Cochrane systematic review evaluated the effectiveness of exogenous steroid sex hormones, specifically testosterone, in treating lower limb atherosclerosis. Despite analyzing three trials involving 109 subjects with intermittent claudication or critical leg ischemia, the review found no significant improvement in the objective measures or subjective symptom relief with testosterone therapy, suggesting no current evidence supporting its efficacy in this condition [156]. However, the authors noted the limited number and dated nature of the trials as potential reasons for the inconclusive findings. Another study investigated the association of endogenous sex hormone levels with extra-coronary calcification (ECC) in a cohort of 2737 postmenopausal women and 3130 men from the Multi-Ethnic Study of Atherosclerosis (MESA). They found that in men, lower free testosterone levels were linked to the increased prevalence and progression of mitral annular calcification (MAC) and descending thoracic aortic calcification (DTAC), even after adjusting for CVD risk factors [157]. Their findings may enhance the idea that testosterone may play a role in vascular calcification patterns, highlighting the potential sex-specific influences on cardiovascular health beyond traditional risk factors. Son et al. demonstrated that testosterone and dihydrotestosterone inhibit inorganic phosphate-induced calcification of human aortic vascular smooth muscle cells by activating the androgen receptor, which in turn promotes the transcription of growth arrest-specific gene 6 (Gas6) via directly binding to its promoter region, revealing a mechanistic link underlying the cardioprotective effects of androgens against vascular calcification [158]. On the other hand, the potential of progesterone to counteract estrogen’s cardioprotective effects has been a contentious topic, with its impact remaining ambiguous and requiring further clarification through additional research [159,160]. Lower levels of progesterone were significantly correlated with more advanced stages of retinal atherosclerotic alterations, while weaker associations were observed for DHEAS and E2 levels, suggesting that low progesterone and possibly low DHEAS and E2 levels might contribute to the development of retinal-artery atherosclerosis in men [161]. Low-testosterone African men with elevated E2 levels showed a higher cardiovascular risk, including hypertension and an increased albumin-to-creatinine ratio, in a study involving participants from multiple ethnic groups [162]. Similar trends were observed among low-testosterone white men, albeit less pronounced [162].

## 4. Sex Hormones and Obesity

Obesity is a multifactorial condition, with one largely overlooked factor being sex/gender. Women, as opposed to men, generally have a higher prevalence of obesity, although it changes by region [163]. Sex hormones such as estrogen and testosterone have a strong effect on fat distribution and metabolism, influencing the tendency toward obesity in different genders. In 2022, 2.5 billion adults worldwide were overweight, which corresponds to 43% of adults, up from 25% of adults in 1990 (WHO). This rising prevalence of obesity has become a major public health concern due to its systemic effects and related chronic complications.

Abdominal obesity in particular is associated with metabolic syndrome (MetS), which is characterized by blood lipid disorders, inflammation, insulin resistance (IR), and other irreversible effects [164]. A meta-analysis study in 2022, including more than 28 million individuals, revealed that the global prevalence of MetS varies between 12.5% and 31.4% across different populations [165]. It is associated with a two-fold increase in the risk of coronary heart disease and cerebrovascular disease, and a 1.5-fold increase in the risk of all-cause mortality [166].

Olivares et al. demonstrated reproductive axis dysfunction and hypogonadal hypogonadism in long-term high-fed mice that exhibited a metabolic profile compatible with IR and MetS, suggesting the strong connection between these pathologies [167]. The influence of sex hormones on these conditions further complicates the picture and emphasizes the need for gender-tailored interventions and screening.

### 4.1. Sex Hormones and Hyperlipidemia

Hyperlipidemia is a main component of MetS [166]. The effects of sex hormones on hyperlipidemia are most evident when evaluating clinical trials with testosterone hormone therapy (THT) and HRT and animal models. Long-term testosterone therapy in hypogonadal men resulted in a decrease in the triglyceride levels [168]. Testosterone inhibits adipocyte LPL activity, a key enzyme that breaks down circulating triglyceride (TG) into absorbable free fatty acids, which are taken up into the fat cells and converted back into TG for storage. Testosterone increases the number of βAR, which, in turn, promotes lipolysis and reduces fatty acid synthesis [169].

Conversely, low testosterone as a result of high aromatase activity leads to a cycle that promotes an increasing adipocyte number and fat deposition, which gradually leads to lower testosterone levels and ultimately increased TG [170]. Evidence from androgen receptor (AR) knockout mouse models demonstrates that the deficiency of androgen action decreases lipolysis and is mainly responsible for the induction of obesity [171].

It was observed that lipoproteins, including TG, were significantly increased following menopause [172]. HRT ameliorates this change by lowering the TG levels [173]. This positive effect of estrogen on the TG levels may be due to its direct hepatic effects through its ERα. Studies with ERα knockout mice demonstrated the ability of estrogen to reduce liver steatosis by reducing the TG levels by acting directly in the liver through ERα [174]. Both estrogen and testosterone decrease the TG levels and reduce fat storage and obesity.

Secretions of adipocytes, such as leptin, adiponectin, and resistin, and gut peptides, such as ghrelin, are considered to be crucial in the interaction between energy homeostasis and reproduction [175]. Obesity is associated with hyperleptinemia. Donahoo et al. demonstrated the effect of daily leptin injections in leptin-deficient mice. The leptin-treated mice exhibited increased LPL activity and weight loss as well as decreased postprandial fatty acids. This suggests the role of leptin in lipid oxidation and decreased adipose tissue lipid storage [176]. Isidori et al. showed the inverse relation between circulating leptin and the free and total testosterone levels, despite adjustments for SHBG, LH, and Ε2 being made [177]. This may be explained by the leptin-associated increases in the estrogen levels that further increase aromatase activity [178] (Figure 1).

### 4.2. Sex Hormones and Hypercholesterolemia

Clinical trials of HRT and TRT demonstrate the positive effects of testosterone and estrogen on the cholesterol levels. The postmenopausal lipoprofile is characterized by increased lipoproteins, TC, LDL, and TC to HDL ratio [172]. Nie et al. concluded that oral HRT significantly decreases the total cholesterol (TC) levels, low-density lipoprotein cholesterol (LDL-C) levels, and TC to high-density lipoprotein cholesterol (HDL-C) ratio compared with placebo or no treatment [173]. It was shown in many studies that all types of oral HRT in postmenopausal women result in a significant reduction in the lipoprotein (a) concentrations compared to the placebo and no treatment [179]. However, there was no accountable difference in the HDL-C levels, which can be contrasted with the decreased level of HDL in male hypogonadism [172]. Studies with tamoxifen, a selective estrogen receptor modulator, revealed that it can affect the lipid profile in females by decreasing TC, LDL-C, and HDL-C. These reductions are dose-dependent [180]. Studies with androstenedione supplementation showed increased E2 levels and ultimately lower triglycerides and HDL-C [181].

Long-term testosterone therapy in hypogonadal men has shown improvements in the lipid profiles, causing a decrease in the total cholesterol but an increase in the TC with a slight increase in the HDL cholesterol accompanied by a decrease in the non-HDL cholesterol and remnant cholesterol [182,183].

### 4.3. Sex Hormones and Insulin Resistance

IR is defined as the inability of circulating insulin to regulate the uptake and utilization of glucose. Insulin also regulates lipid metabolism in hepatic and adipocyte cells. IR can increase lipogenesis, resulting in the development of non-alcoholic fatty liver disease, which is seen as a fundamental component of MetS [184]. Clinical trials of HRT treatment showed a significant reduction in the HOMA-IR score and new-onset diabetes in women without diabetes. In postmenopausal women with diabetes, HRT reduced the fasting blood glucose (FBG) [185]. This effect may be due to a decrease in visceral adipose tissue. Thorne et al. established that surgical removal of omental fat tissue resulted in long-term improvements in the metabolic profile with a 2–3-fold improvement in glucose tolerance and insulin sensitivity, with decreased FBG and insulin levels compared to control subjects [186]. Estrogen treatment in male mice on an obesogenic diet prevented diet-induced increases in adipose tissue and improved glucose–insulin homeostasis [187].

Long-term testosterone treatment in hypogonadal men resulted in a decrease in FBG and a progressive decline of HbA1c; the opposite holds true for untreated men [168]. ADT in prostate cancer patients has been shown to have a negative effect on glycemic control and HbA1c levels as well as the increased incidence of new-onset diabetes [188]. After 6 months of testosterone therapy in idiopathic hypogonadotropic patients, there was a dramatic decrease in the HOMA-IR score as well as in the body fat mass, with a concurrent increase in the BMI and body lean mass [189].

A growing body of evidence suggests a correlation and causative effect of inflammation on insulin resistance. The proinflammatory cytokine TNF-α has been demonstrated to mediate insulin resistance as a result of obesity in many rodent obesity models [190]. TNF-α was overexpressed in white adipose tissue (WAT) in obese and insulin-resistant states; mice lacking the TNF-α ligand were relatively protected from obesity-induced insulin resistance [191] (Figure 1).

### 4.4. Sex Hormones and Inflammation

Adipose tissue has a highly vascularized connective tissue matrix containing a multitude of immune cells, most notably macrophages [192]. Transcript expression in perigonadal tissue in mice of obesity-related mutations revealed that of the 100 most significant mutations, 30% were macrophage-related. Immunohistochemical analysis of this tissue and human subcutaneous tissue highlights the importance of macrophages by positively correlating the percentage of F4/80+ cells, a macrophage marker, with the adipose size and body mass. The normal percentage of macrophages in adipose tissue is 10%, but it can reach 50% in obesity [193]. Adipose tissue macrophages are responsible for TNF-α expression and subsequent increases in iNOS and IL-6 expression [194]. Immunological studies show that abdominal and omental WAT secrete 2–3 times more IL-6 than subcutaneous WAT [195]. In vitro studies show that estrogen implements anti-inflammatory effects through ERα, which are expressed in immune and cytokine-releasing cells, by directly decreasing the number of proinflammatory cytokines [196]. These proinflammatory mediators also directly affect cellular metabolism by decreasing insulin sensitivity and increasing lipolysis and hepatic triglyceride secretion [193,194].

Clinical trials have shown considerable variations in inflammatory markers with sex hormone treatments. For example, oral conjugated equine estrogen increased the CRP levels by 48% after 6 months and by 64% after 12 months [197]. The research is conflicting, with exogenous estrogen increasing the CRP levels and endogenous estrogen decreasing the CRP levels [198]. Conversely, long-term testosterone treatment showed a decline in the CRP levels in all patients, regardless of the baseline weight [168]. In men with pre-existing MetS, the calculated free testosterone and SHBG were 11% and 18% lower. Men with low testosterone levels were 2.7 times more likely to have MetS in the age-adjusted analysis. The total and free testosterone and SHBG were inversely associated with the concentrations of insulin, glucose, triglycerides, CRP, and CRP-adjusted ferritin and positively associated with HDL-C [199].

### 4.5. Sex Hormones and Fat Accumulation & Distribution

WAT can be broadly localized as visceral and subcutaneous. Premenopausal women have an affinity toward accumulating subcutaneous fat, which was shown to have metabolically protective effects. In contrast, men often accumulate visceral fat, which largely contributes to metabolic dysregulation and disease. Postmenopausal women accumulate visceral fat while decreasing subcutaneous depots. These gender-specific differences in adipose storage sites are important in understanding the effect of sex hormones on adiposity and metabolic consequences [200].

Reduced total testosterone is frequently observed in men with abdominal obesity and MetS [201]. Men with lower baseline total T levels were at higher risk of developing MetS, even after adjustment for the SHBG, BMI, or HOMA-IR. This demonstrates the fundamental role of low T concentrations as a causative factor in the development of MetS, leading to an increased risk of at least 40–70% compared to eugonadal men. Not only does obesity directly impact the testosterone levels, but reduced testosterone levels contribute to increased adiposity [202]. Conversely, long-term testosterone therapy in hypogonadal men produces progressive and sustained improvements in the body weight, waist circumference, and body mass index, regardless of the weight at baseline [182,183,201]. In women with polycystic ovarian syndrome, pathologic androgen excess, especially increased free testosterone levels, was positively correlated with increased abdominal adiposity and visceral fat tissue accumulation, whereas the SHBG levels were negatively correlated with abdominal fat [201].

Like testosterone, estrogen levels and visceral fat accumulation are inversely related. Low estrogen levels induced by deletion in aromatase in mice result in an obesity–insulin-resistant phenotype, similar to findings in ovarian failure [203]. High estrogen levels have been shown to increase subcutaneous fat and diminish visceral fat expansion [204]. This may be a result of the direct effect of estrogen as progesterone, androgen, and estrogen receptors are found in adipose tissue. Visceral adipose tissue has a higher concentration of AR, whereas subcutaneous tissue has higher ER and PR expression [205]. Adipose tissue-specific AR knockout mice have increased intra-adipose E2 levels, which leads to pronounced subcutaneous obesity and hyperleptinemia [206]. Estrogen regulates the body fat distribution and adiposity through ERα and ERβ receptors [207]. ERα has been reported to have a stronger effect on the energy balance and expenditure. Heine et al. proved that male and female mice with the total deletion of ERα have increased adiposity, with a two-fold increase in visceral fat depots, suggesting the inhibitory effect of estrogen on adipose tissue [208]. The postmenopausal decline in circulating ERα is causative in terms of increased visceral adiposity, which is improved with ERα replacement [209].

## 5. Ongoing Clinical Trials and Future Directions

Clinical trials investigating the impact of sex hormones on cardiorenal and metabolic health involve diverse populations and utilize a range of interventions, including aromatase inhibitors, selective estrogen receptor modulators, testosterone/estrogen replacement therapy, GnRH analogs, anti-androgens, as well as GAHTs. While GAHT has been explored in previous clinical trials, there is a growing interest in the subject. Currently, there is a notable increase in ongoing clinical trials that specifically examine the effects of gender-affirming (cross-sex) hormone therapies on various cardiorenal and metabolic parameters. These investigations encompass a wide range of factors, including cardiovascular risk factors, insulin sensitivity, beta-cell function, body composition, anthropometric parameters, and fat content in organs such as the myocardium, liver, and pancreas. This topic has been a focus of past research and continues to be explored in multiple ongoing trials [210,211,212,213,214,215] These investigations are valuable for comprehensively understanding the long-term impacts and potential risks associated with GAHTs. Given the growing access to and awareness of these treatments in today’s world, such research not only aids in gauging their safety and efficacy but also enhances our understanding of how sex hormones influence cardiometabolic parameters. In another clinical trial, by inducing an elevated myocardial triglyceride content through fasting and utilizing advanced imaging techniques, such as cardiac magnetic resonance imaging and spectroscopy, researchers aim to elucidate any sex-specific differences in cardiac morphology and function. Additionally, the study delves into whether estrogen offers a protective mechanism against myocardial steatosis-induced dysfunction by modulating ovarian sex hormones with a GnRH antagonist and subsequent estrogen add-back. Ultimately, this research promises insights into the underlying mechanisms and potential therapeutic avenues for addressing sex-specific variations in myocardial steatosis-related left ventricular dysfunction [216]. Furthermore, there are observational studies investigating the level of various sex hormones and their relationship with metabolic parameters. For instance, an ongoing trial, the FEMAIL study, aims to investigate the association between 11-oxygenated androgens and the metabolic risk, particularly focusing on their impact on glucose metabolism and the type 2 diabetes risk in women, including those with polycystic ovary syndrome [217]. Utilizing advanced techniques like liquid chromatography–tandem mass spectrometry for multi-steroid profiling and non-targeted serum metabolomics, along with clinical questionnaires, anthropometric assessments, and muscle biopsies, the study seeks to elucidate the role of these androgens as potential biomarkers and novel metabolic risk factors.

As the comprehension of sex hormone dynamics and their impact on cardiorenal and metabolic health progresses, numerous promising avenues invite exploration (Table 1). Primarily, longitudinal inquiries spanning diverse demographics are imperative to grasp the enduring ramifications of sex hormone interventions on cardiovascular outcomes, renal function, and metabolic well-being throughout various life phases. Particularly, as the realm of gender-affirming healthcare advances, ongoing investigations should prioritize discerning the nuanced effects of GAHTs on cardiorenal and metabolic parameters, ensuring inclusive and thorough healthcare for transgender and gender-diverse individuals. Furthermore, given the increased prevalence of adverse cardiometabolic outcomes in postmenopausal females, future clinical trials should take into account the changing physiological processes during this period and consider different hormone concentrations and dosages in line with patient characteristics. The current global epidemic of obesity has been shown to have a causal relationship with many cardiometabolic and renal disorders, but the role of sex hormones in its occurrence and their potential use in its treatment need further investigation. Experimental animal studies are of the utmost importance for the comprehensive understanding of sex hormones and their interaction with cardiac, renal, and metabolic processes. Evaluating and comparing the effects of androgens and estrogens on both sexes and in different age and weight groups are important next steps in this realm of research, which will be of help in designing both gender-affirming and postmenopausal hormone therapies as well as exploring their roles in obesity. Enhanced insight into sex hormone biology and its consequences for cardiorenal and metabolic health will lay the groundwork for more precise and efficacious therapeutic approaches in the future. The rise of personalized medicine presents opportunities for customized HRT, considering individual genetic predispositions and hormone receptor profiles. Additionally, the integration of state-of-the-art technologies such as single-cell transcriptomics and epigenetic profiling holds promise in uncovering novel molecular pathways governing sex hormone-mediated responses in the cardiorenal and metabolic domains.

The complex relationship between cardiovascular and renal systems, which are extensively influenced by the varying levels of sex hormones, requires novel treatment modalities that take the individual characteristics of the patients into account. The current approach to this syndrome includes aggressive lifestyle modification measures that place an emphasis on diet, physical activity and behavioral modification, as well as medical management, including sodium-glucose costransporter-2 inhibitors (SGLT2i) and glucagon-like peptide-1 receptor agonists (GLP-1 RA) [218,219,220]. These two medications are especially important in relation to CKM syndrome as they have cardiorenal protective effects, in addition to ensuring glycemic control. Nevertheless, these approaches are far from being adequate, and personalized treatment plans that are monitored by an integrated multidisciplinary cardio-renal-metabolic care team are needed for the effective management of patients with CKM syndrome. In this regard, the formation of a cardiorenal subspecialty has been recommended [221]. We suggest that endocrine processes, especially the varying levels of sex hormones and their effects on cardiorenal and metabolic outcomes, should be an integral part of the curriculum of this subspecialty. This way, specialists will be able to provide efficient and harmonized care to their patients of varying ages and from varying backgrounds by making the best use of emerging treatment options.

## 6. Conclusions

Understanding the intricate relationship between sex hormones and cardiac, renal, and metabolic health is crucial for tailoring interventions to individual needs. The complex interplay between sex hormones and these physiological systems impacts various aspects of health. To develop effective interventions, it is essential to grasp how hormonal alterations influence cardiac function, renal health, and metabolism. Additionally, awareness of the potential effects of treatments targeting sex hormones is vital, as they can significantly impact overall health outcomes. While estrogen demonstrates protective effects against renal injury and influences nephron structure and function, testosterone’s influence suggests gender-specific mechanisms underlying the pathogenesis of CKD. Similarly, moving to cardiac health, estrogen emerges as a key player in mitigating atherosclerosis and vascular injury, exerting effects such as enhanced NO production and arterial dilation, whereas testosterone therapy tends to decrease inflammation, impacting vascular remodeling and the expression of adhesion molecules and growth factors. Overall, these findings emphasize the differential effects of sex hormones on cardiorenal health, which might necessitate gender-tailored interventions. Furthermore, sex hormones also profoundly impact metabolic health, particularly in the context of obesity and MetS. Estrogen and testosterone influence fat distribution and metabolism, contributing to gender-specific differences in the obesity prevalence. HRTs show promise in improving lipid profiles and insulin sensitivity, thus mitigating the risk of MetS and type 2 diabetes. Understanding these hormonal influences on metabolic health is crucial for developing personalized approaches to obesity management and metabolic disorders. Overall, recognizing the impact of sex hormones on cardiac, renal, and metabolic health is essential for advancing personalized medicine and improving outcomes in patients with metabolic and cardiovascular diseases. By acknowledging the nuanced interplay between sex hormones and these systems, clinicians and researchers can develop more effective prevention and management strategies tailored to individual hormonal profiles, ultimately improving patient care and outcomes.

## Figures and Tables

**Table 1:** Knowns, unknowns, and future research directions in terms of sex hormones and their influence on cardiorenal, vascular, and metabolic health.

	Knowns	Unknowns	Future Research Recommendations
**Renal health**	CKD exhibits a higher prevalence in women compared to males, despite a larger proportion of males receiving nephrological care. Female CKD patients have less likelihood of progressing to ESRD and lower risks of CV events and mortality. Gender differences in CKD are influenced by oxidative stress, inflammation, apoptosis, and renal hemodynamics, with estrogen providing protective effects and testosterone exacerbating renal injury. Transgender hormone therapies impact kidney health, with significant changes observed in serum creatinine levels, particularly in transgender women.	The specific mechanisms underlying the gender disparities observed in CKD prevalence, progression to ESRD, and treatment outcomes. The comprehensive effects of gender-affirming hormone therapy on kidney function. The long-term effects of testosterone therapy on renal function in transgender men. The detailed molecular pathways through which estrogen and testosterone modulate oxidative stress, inflammation, apoptosis, and renal hemodynamics in CKD.	Conduct longitudinal studies to evaluate the long-term effects of GAHTs on renal function in transgender individuals. Explore the potential mechanisms provoking changes in renal function during testosterone therapy of transgender men, develop strategies for mitigating risks. Investigate the underlying molecular mechanisms driving gender disparities in CKD prevalence, progression, and treatment outcomes to develop personalized treatments. Elucidate the molecular pathways through which estrogen and testosterone influence oxidative stress, inflammation, apoptosis, and renal hemodynamics in CKD.
**Cardiac health**	Estrogen inhibits cardiomyocyte hypertrophy, enhances mitochondrial activity, prevents apoptosis, promotes cardiac regeneration, and modulates cardiac electrical conductance, potentially contributing to a lower incidence of HF and delayed onset of IHD in women. Androgens have pro-hypertrophic effects, induce cardiomyocyte apoptosis, and increase oxidative stress, possibly contributing to a higher likelihood of HF in males and earlier onset of IHD.	AR subtypes on the surface of and inside cardiomyocytes. The relationship between hormonal imbalances and the development and progression of HF, as well as the impact of HF on hormonal imbalances. The reasons behind the delayed onset of IHD in women compared to males and the potential molecular mechanisms underlying estrogen’s cardioprotective effects.	Clinical trials to assess the CV effects of HRT in postmenopausal women, considering different hormone formulations, dosages, and patient characteristics. Advanced preclinical models to study sex-specific cardiac responses and test potential therapeutic interventions. Elucidate the molecular mechanisms underlying estrogen’s cardioprotective effects against IHD and investigate potential therapeutic interventions.
**Vascular health**	Premenopausal women have lower blood pressure and lower incidence of ASCVD compared to age-matched men, but the prevalence increases in postmenopausal women. Estrogen promotes vasodilation and vascular remodeling, decreases migration and proliferation of VSMC. It increases the RAS components that oppose increased blood pressure and enhances βAR-induced vasorelaxation in SNS. It also controls lipid metabolism, inflammation, and plaque stability to mitigate ASCVD progression. Androgens may provoke both vasodilation and vasoconstriction. They increase the vasoconstrictor components of RAS and promote αAR-induced vasoconstriction.	AR subtypes on the surface of and inside vascular cells. Detailed molecular pathways through which estrogen and androgens modulate vascular cell function, macrophage polarization, endothelial function, and plaque stability. The role of progesterone in CV health and its potential to counteract estrogen’s cardioprotective effects. Research on other sex hormones, such as DHEA-S, and their influence on atherosclerosis pathophysiology and clinical outcomes.	Include diverse populations in future clinical trials of ASCVD and HTN and consider stratification by sex, age, menopausal status, and CV risk factors. Studies on the long-term atherosclerotic and hypertensive effects of GAHTs in transgender individuals, stratifying by hormone regimen, duration of treatment, and other health factors. Advanced preclinical models to investigate sex-specific vascular respons es in HTN and ASCVD. Elucidate molecular mechanisms underlying the effects of estrogen and androgen on vascular cells in HTN and ASCVD.
**Obesity**	Testosterone is associated with lower triglyceride levels and estrogen potentially lowers total cholesterol levels. Sex hormones influence adipose tissue distribution as men tend to accumulate visceral fat and women subcutaneous fat. Testosterone and estrogen treatments influence insulin sensitivity in animal models.	Exact molecular mechanisms underlying sex hormones’ effects in obesity-related conditions. The long-term effects of sex hormone therapies on metabolic health and CV outcomes. Variability in response to sex hormone therapies among individuals. Potential role of gut microbiota in mediating the effects of sex hormones on obesity and metabolic health.	Investigate the mechanisms underlying the sex-specific differences in fat distribution and its implications for metabolic health. Investigate the interaction between sex hormones and genetics, environmental factors in obesity and metabolic syndrome. Evaluate the efficacy and safety of gender-tailored interventions for obesity prevention and management.

**Abbreviations:** CKD, chronic kidney disease; ESRD, end-stage renal disease; CV, cardiovascular; GAHT, gender-affirming hormone therapy; HF, heart failure; IHD, ischemic heart disease; HRT, hormone replacement therapy; AR, androgen receptor; VSMC, vascular smooth muscle cell; HTN, hypertension; ASCVD, atherosclerotic cardiovascular disease; RAS, renin–angiotensin system; αAR: α-adrenergic receptor; βAR: β-adrenergic receptor; SNS, sympathetic nervous system; DHEA-S, dehydroepiandrostenedione sulfate.
